# Light regimes differentially affect baseline transcript abundance of stress-axis and (neuro)development-related genes in zebrafish (*Danio rerio*, Hamilton 1822) AB and TL larvae

**DOI:** 10.1242/bio.028969

**Published:** 2017-10-05

**Authors:** Ruud van den Bos, Jan Zethof, Gert Flik, Marnix Gorissen

**Affiliations:** Radboud University, Institute for Water and Wetland Research, Department of Animal Ecology and Physiology, Heyendaalseweg 135, 6525 AJ Nijmegen, The Netherlands

**Keywords:** Larvae, Light regimes, Gene expression, Development, Stress axis

## Abstract

Many strains of zebrafish (*Danio rerio*) are readily available. Earlier we observed differences between AB and Tupfel long-fin (TL) larvae regarding baseline hypothalamus-pituitary-interrenal (HPI) axis activity and (neuro)development. Light regimes, i.e. 14 h light:10 h dark and 24 h continuous dark or light, affect hatching rate and larval growth. Here, we assessed baseline transcript abundance of HPI-axis-related genes and (neuro)development-related genes of AB and TL larvae (5 days post fertilisation) using these light regimes. A principal component analysis revealed that in AB larvae the baseline expression of HPI-axis-related genes was higher the more hours of light, while the expression of (neuro)development-related genes was higher under 14 h light:10 h dark than under both continuous light or dark. In TL larvae, a complex pattern emerged regarding baseline expression of HPI-axis-related and (neuro)development-related genes. These data extend data of earlier studies by showing that light regimes affect gene-expression in larvae, and more importantly so, strengthen the notion of differences between larvae of the AB and TL strain. The latter finding adds to the growing database of phenotypical differences between zebrafish of the AB and TL strain.

## Abstract (precis)

**Summary:** This study shows gene expression levels of zebrafish AB and TL larvae differ in relation to light regimes, strengthening earlier observations: AB and TL are not interchangeable strains.

## INTRODUCTION

Zebrafish (*Danio rerio*, Hamilton 1822) have become a popular model organism in biomedical research (Stewart et al., 2014). Many strains are readily available, which have been shown to strongly differ in phenotype and/or genotype (Guryev et al., 2006; Stewart et al., 2014). Systematically genotyping and phenotyping strains is therefore critical to enhance reproducibility of experiments both within and between laboratories. We recently showed that larvae [day 5 post fertilisation (5 dpf)] of the AB and Tupfel long-fin (TL) strain differ in baseline hypothalamus-pituitary-interrenal (HPI) axis activity, the expression of (neuro)development-related and (innate) immune system-related genes, as well as light-dark motor behaviour (van den Bos et al., 2017). Here, we extend these data by studying the effects of different light regimes on baseline expression of HPI-axis-related genes and (neuro)development-related genes.

Among laboratories, embryos and larvae have been, and still are, reared under different light conditions: e.g. 14 h light:10 h dark (14L:10D), continuous (24 h) dark and continuous (24 h) light. While 14L:10D is the most relevant light regime ecologically (Spence et al., 2008), other regimes are used for ease. Different light regimes lead to differences in hatching rate, growth, light-dark motor behaviour and the occurrence of malformations (Ahmad, 2014; Villamizar et al., 2014). Until now it is not clear how these different light regimes affect baseline expression of HPI-axis-related genes [*corticotropin-releasing factor* (*crf*), *corticotropin-releasing factor binding protein* (*crf-bp*), *mineralocorticoid receptor* (*mr*; *nr3c2*), *glucocorticoid receptor alpha* (*gr-alpha*; *nr3c1α*), and *glucocorticoid receptor beta* (*gr-beta*; *nr3c1β*); van den Bos et al., 2017] and (neuro)development-related genes [*proliferating cell nuclear antigen* (*pcna*); *brain-derived neurotrophic factor* (*bdnf*); *neuronal differentiation factor 1* (*neurod1*); *insulin-like growth factor 1* (*igf1*); *growth hormone 1* (*gh1*); *cocaine- and amphetamine-regulated transcript 4* (*cart4*); van den Bos et al., 2017] and whether this is similar in different strains. Hence, we assessed the effects of different light regimes hereon in 5 dpf AB and TL larvae.

## RESULTS

Table 1 shows the expression levels of the different genes as well as the statistics for AB and TL larvae reared under different light regimes. Two important findings emerged. First, for all genes, but *gh1*, significant interaction effects were found. This indicates that light regimes had a different effect on gene-expression in AB and TL larvae. Hence, we decided to pay no attention to significant differences between light regimes per se (observed for all genes but for *gh1* and *cart4*) as they mask light-regime-induced differences between strains. Second, the data in Table 1 show that across light regimes several genes display similar changes in either AB or TL larvae. This suggests that the expression levels of these genes may be interrelated across light regimes in AB or TL larvae. Based on these two findings, we decided to analyse data strain-wise using principal component analysis (PCA) to assess how expression patterns of genes interrelate across light regimes, excluding *gh1* as it showed no significant effects.

**Table 1.**
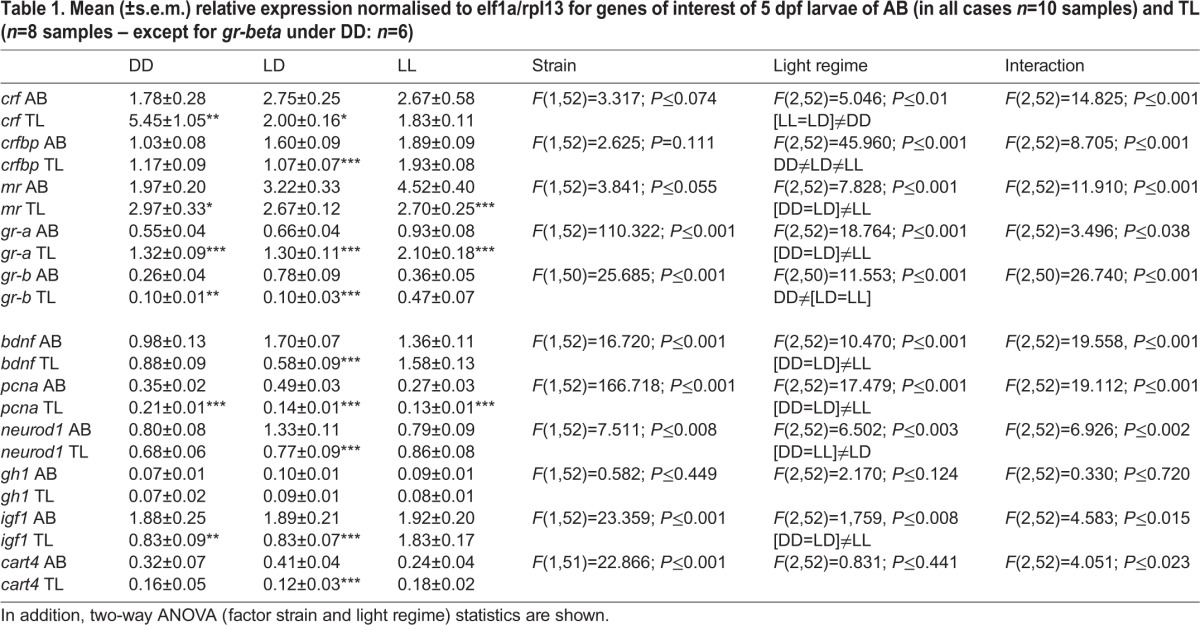
**Mean (±s.e.m.) relative expression normalised to elf1a/rpl13 for genes of interest of 5 dpf larvae of AB (in all cases *n*=10 samples) and TL (*n*=8 samples – except for *gr-beta* under DD: *n*=6)**

### AB strain

The Kaiser-Meyer-Olkin (KMO) value was sufficiently high (0.605), while Bartlett's test of sphericity was significant (*n*=30; Χ^2^=191.671, d.f.=45, *P*≤0.001); both are measures to assess whether it is appropriate to run factor analysis (see Materials and Methods), thereby implying meaningful PCA results (Table 2A). Three factors were found, explaining 74.5% of variance: factor 1: HPI-axis (*crf*, *crf-bp*, *mr*, *gr-alpha*); factor 2: general (neuro)development (*gr-beta*, *bdnf*, *pcna*, *neurod1*, *cart4*); and factor 3: growth (*igf1*).

**Table 2.**
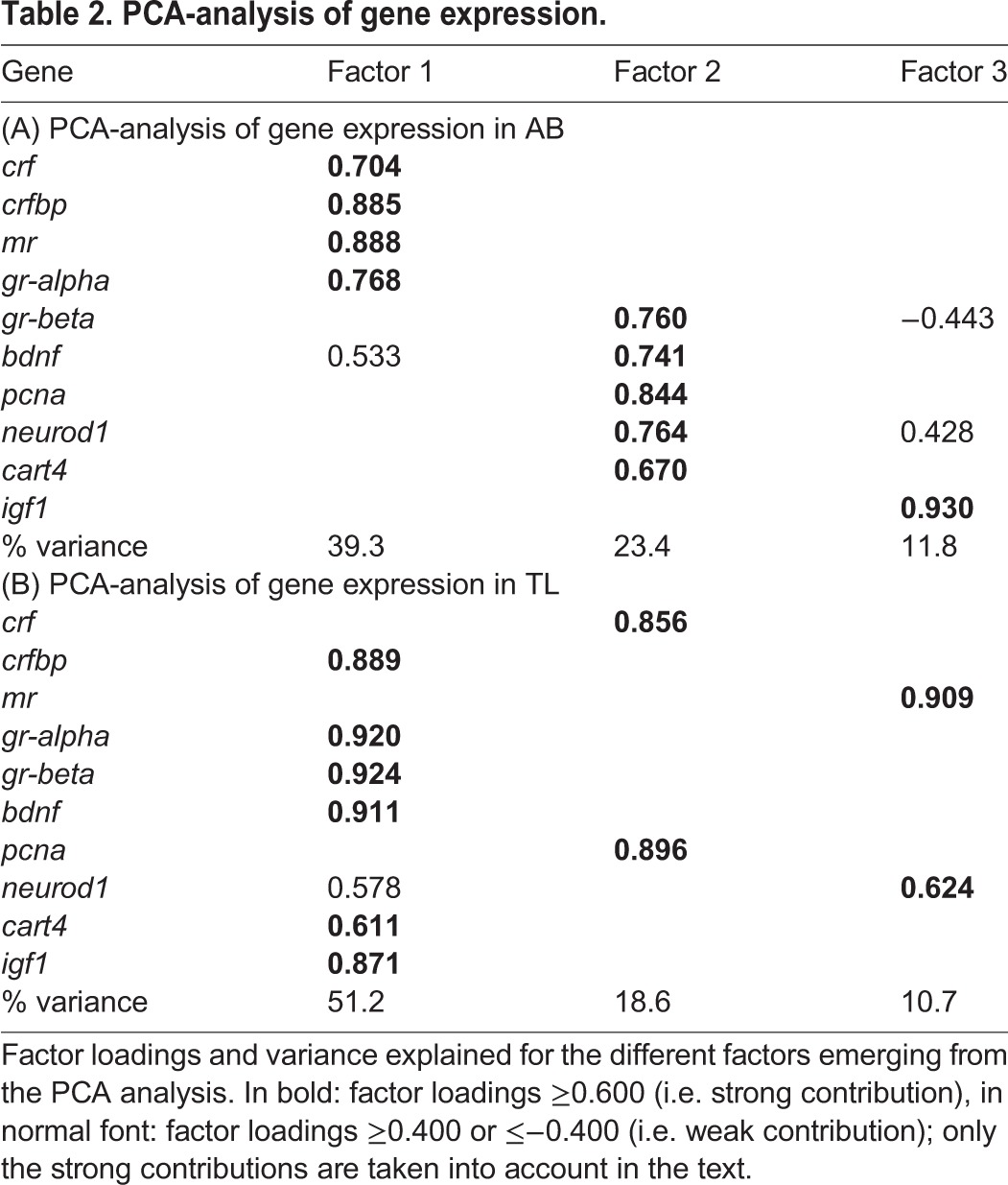
**PCA-analysis of gene expression.**

**Fig. 1.**
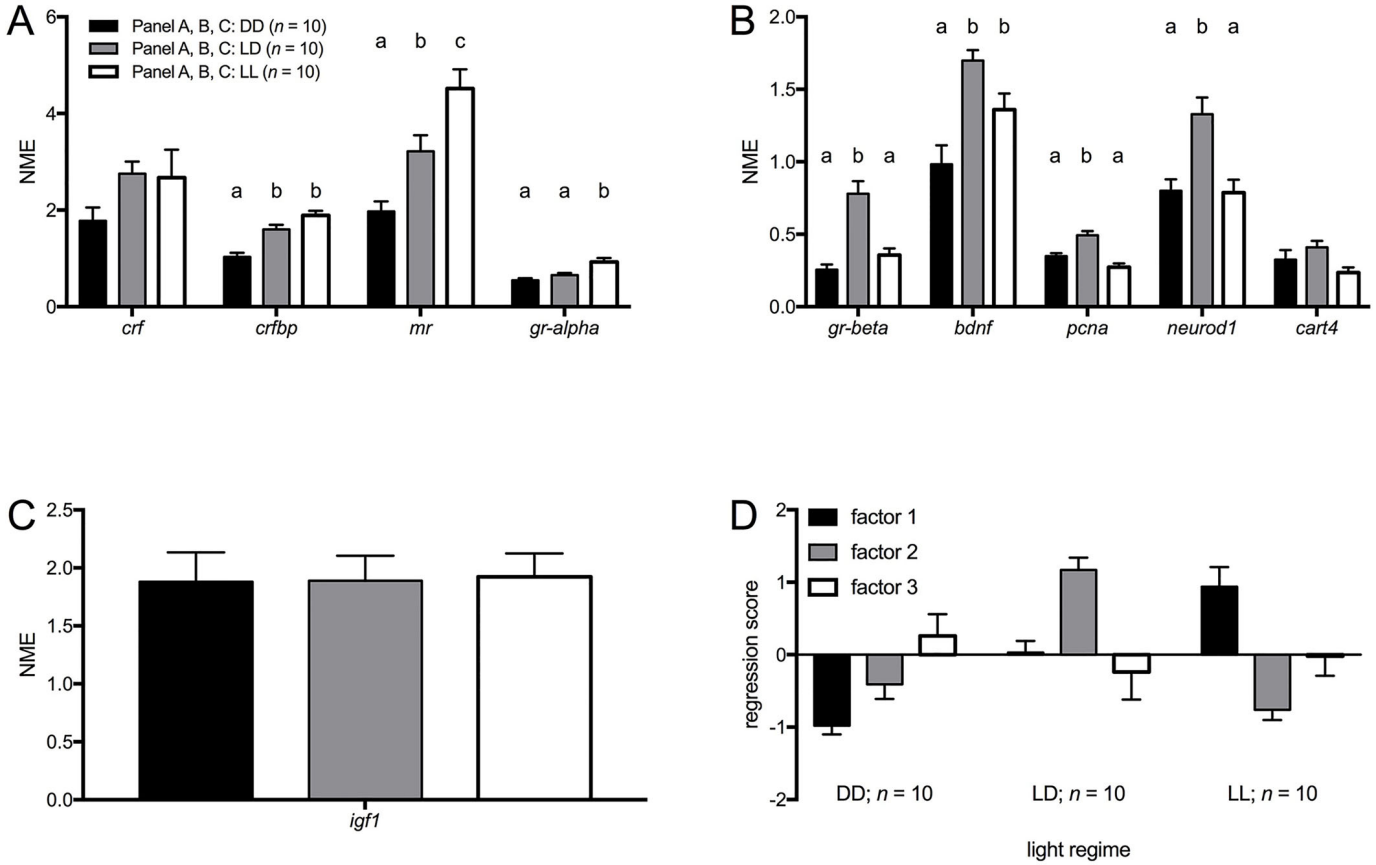
**Baseline transcript abundance in AB larvae under different light regimes.** (A) Baseline transcript abundance [normalised mean expression (NME)+s.e.m.] of genes that loaded on factor 1 (HPI-axis) of the PCA. (B) Baseline transcript abundance [normalised mean expression (NME)+s.e.m.] of genes that loaded on factor 2 [(neuro)developmental genes] of the PCA. (C) Baseline transcript abundance [normalised mean expression (NME)+s.e.m.] of *igf1* (loaded on factor 3; growth) of the PCA. (D) Regression scores (mean+s.e.m.) of factors from the PCA. Different letters indicate significant differences (Tukey HSD, *P*≤0.05) between light regimes following a significant one-way ANOVA.

Apart from *gr-beta*, all genes classically related to the HPI-axis (*crf*, *crfbp*, *mr* and *gr-alpha)* loaded onto the same factor. In general, expression levels of HPI-axis-related genes increased from 24 h darkness condition (DD) to 24 h light condition (LL) (Fig. 1A) [one-way ANOVA: *crf F*(2,27)=1.845, n.s.; *crfbp F*(2,27)=22.875, *P*≤0.001; *mr F*(2,27)=15.737, *P*≤0.001; *gr-alpha F*(2,27)=11.454, *P*≤0.001], confirmed when factor 1 scores were analysed (Fig. 1D): LL values were significantly higher than those of 14L:10D (LD) and DD, and LD values were significantly higher than those of DD [one-way ANOVA: *F*(2,27)=23.811, *P*≤0.001; Tukey HSD: *P*≤0.05]. Thus, the more hours of light per day, the higher the baseline expression of *crf*, *crfbp*, *mr* and *gr-alpha*, suggesting up-regulation of baseline HPI-axis activity.

*Gr-beta* loaded onto the same factor as (neuro)development-related genes, i.e. *bdnf*, *pcna*, *neurod1* and *cart4.* In general, expression levels of (neuro)development-related genes were higher under LD than under LL or DD (Fig. 1B) [one-way ANOVA: *gr-beta F*(2,27)=21.190, *P*≤0.01; *bdnf F*(2,27)=11.145, *P*≤0.01; *pcna F*(2,27)=19.734. *P*≤0.001; *neurod1 F*(2,27)=10.439, *P*≤0.001; *cart4 F*(2,27)=2.919, n.s.], confirmed when factor 2 scores were analysed (Fig. 1D): LD values were significantly higher than those of LL and DD [one-way ANOVA: *F*(2,27)=35.369, *P*≤0.001; Tukey HSD: *P*≤0.05].

No significant differences were found for *igf1* (Fig. 1C) [one-way ANOVA: *F*(2,27)=0.010, *P*>0.05] or factor 3 scores (Fig. 1D) [one-way ANOVA: *F*(2,27)=0.603, n.s.].

### TL strain

The KMO value was sufficiently high (0.685), while Bartlett's test of sphericity was significant (*n*=26; KMO=0.685; Χ^2^=209.952, d.f.=45, *P*≤0.001), thereby implying meaningful PCA results as indicated above (Table 2B). Three factors were found, explaining 80.5% of variance: factor 1: HPI-axis, growth and development (*crfbp*, *gr-alpha*, *gr-beta*, *bdnf*, *igf1*, *cart4*); factor 2: HPI-axis and cell-proliferation (*crf*, *pcna*); factor 3: HPI-axis and neurodevelopment (*mr*, *neurod1*).

**Fig. 2.**
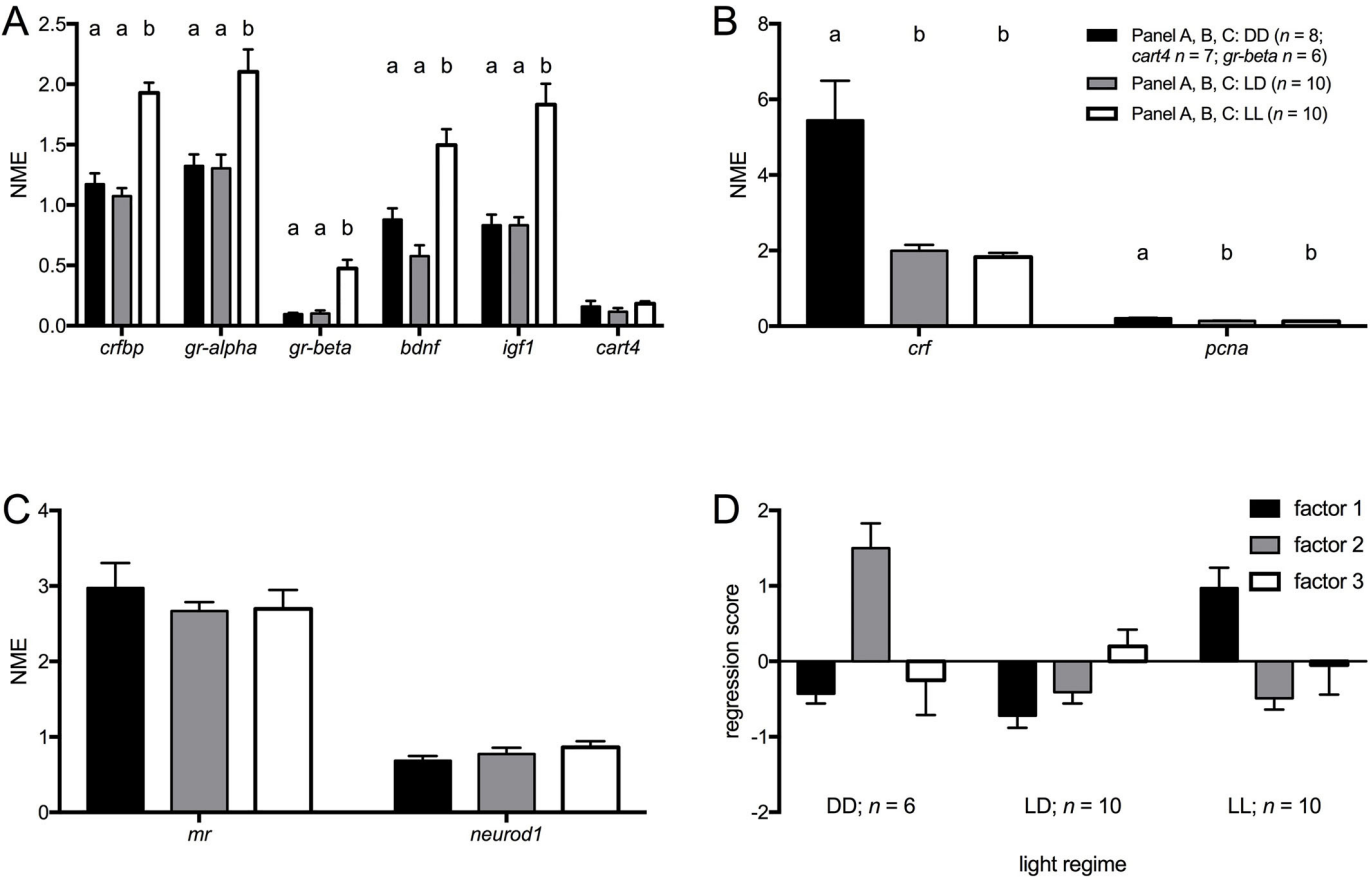
**Baseline transcript abundance in TL larvae under different light regimes.** (A) Baseline transcript abundance [normalised mean expression (NME)+s.e.m.] of genes that loaded on factor 1 [HPI-axis, growth and (neuro)development] of the PCA. (B) Baseline transcript abundance [normalised mean expression (NME)+s.e.m.] of genes that loaded on factor 2 (HPI-axis/cell proliferation) of the PCA. (C) Baseline transcript abundance [normalised mean expression (NME)+s.e.m.] of genes that loaded on factor 3 [HPI-axis and (neuro)development] of the PCA. (D) Regression scores (mean+s.e.m.) of factors from the PCA (Table 2B). letters indicate significant differences (Tukey HSD, *P*≤0.05) between light regimes following a significant one-way ANOVA.

Expression levels of HPI-axis, growth and development-related genes were in general higher under LL than under LD or DD (Fig. 2A) [one-way ANOVA: *crfbp F*(2,25)=35.736, *P*≤0.001; *gr-alpha F*(2,25)=10.633, *P*≤0.001; *gr-beta F*(2,23)=18.723, *P*≤0.001; *bdnf F*(2,25)=19.686, *P*≤0.001; *igf1 F*(2,25)=22.954, *P*≤0.001; *cart4 F*(2,25)=1.291, n.s.], confirmed when factor 1 scores were analysed (Fig. 2D): values of LL were significantly higher than those of LD and DD [one-way ANOVA: *F*(2,23)=19.361, *P*≤0.001; Tukey HSD: *P*≤0.05].

Expression levels of HPI-axis-related and cell proliferation-related genes were higher under DD than under LL or DD (Fig. 2B) [one-way ANOVA: *crf F*(2,25)=13.782, *P*≤0.001; *pcna F*(2,25)=13.019, *P*≤0.001], confirmed when factor 2 scores were analysed (Fig. 2D): values of DD were significantly higher than those of LD or LL [one-way ANOVA: *F*(2,23)=27.450, *P*≤0.001; Tukey HSD: *P*≤0.05].

No significant differences were found for *mr* or *neurod1* (Fig. 2C) [one-way ANOVA: *mr F*(2,25)=0.467, n.s.; *neurod1 F*(2,25)=1.226, *P*>0.05] or factor 3 scores (Fig. 2D) [one-way ANOVA: *F*(2,23)=0.374, n.s.].

## DISCUSSION

The present data show that light regimes affect gene expression levels of genes related to the HPI-axis and (neuro)development, yet differently so in larvae of the AB and TL strain. Across light regimes baseline expression levels of several genes changed in a similar way, and hence seemed interrelated in the way they were affected by different light regimes. Using PCA as a tool to analyse this interrelationship, we observed that in AB larvae, genes were clearly separated along differences in function, HPI-axis and (neuro)development, while it also suggested that *gr-beta* may be involved in (neuro)development. A different and less clear pattern emerged when we analysed the data of TL larvae using PCA.

### AB strain

The more hours of light per day, the higher the baseline expression of *crf*, *crfbp*, *mr* and *gr-alpha*, suggesting up-regulation of baseline HPI-axis activity. The HPI-axis is functional in larvae from day 3 onwards, i.e. following hatching (Alsop and Vijayan, 2008; Alderman and Bernier, 2009; Eto et al., 2014). Whether these changes are related to the increased hatching rate under continuous light and decreased hatching rate under continuous dark compared to 14L:10D (Ahmad, 2014; Villamizar et al., 2014) needs to be determined.

*Gr-beta* loaded onto the same factor as (neuro)development-related genes, i.e. *bdnf*, *pcna*, *neurod1* and *cart4.* Interestingly, it was recently shown that *gr-beta* – independently of *gr-alpha* – may be functionally involved in the development of zebrafish larvae (Chatzopoulou et al., 2015). Thus, these data show that baseline gene expression levels of (neuro)development-related genes were collectively higher under a 14L:10D regime than under either continuous light or dark regimes. This seems in line with observations of decreased growth and more malformations under either continuous light or dark compared to 14L:10D (Villamizar et al., 2014). These data add to the growing awareness that 14L:10D may be the optimal condition for proper (neuro)development of zebrafish (Spence et al., 2008).

The observation that no effect was found for *igf1* seems in line with the fact that also no differences were found for *gh1*. As both genes are involved in growth-related processes [*igf1*; growth, brain development, maturation and neuroplasticity (Eivers et al., 2004; Dyer et al., 2016); *gh1*: synchronisation of somatic growth and energy metabolism (Zhu et al., 2007; McMenamin et al., 2013)], we predicted effects of light regimes on the expression of these genes (Villamizar et al., 2014). Why we observed no effects in the expression levels of these genes warrants further studies.

### TL strain

The expression levels of *crfbp*, *gr-alpha*, *gr-beta*, *bdnf*, *igf1* and *cart4* were interrelated with increased expression levels, especially under continuous light, suggesting up-regulation of baseline HPI-axis activity and accelerated/stronger development (Ahmad, 2014; Villamizar et al., 2014). The expression levels of *crf* and *pcna* were interrelated with increased expression levels especially under continuous dark. We have observed that *pcna* levels decrease from 1 dpf to 5 dpf in larvae (R.v.d.B., J.Z., G.F. and M.G., unpublished data), suggesting slower development under continuous dark (Ahmad, 2014; Villamizar et al., 2014). Preliminary analysis showed that cortisol levels were highest under continuous dark and lowest under continuous light, which would be in line with the high transcript abundance of *crf* under continuous dark, yet at variance with those of *crfbp*, *gr-alpha* and *gr-beta*. It is clear that more studies are needed to clarify these seemingly conflicting data.

### Limitation

We have sampled larvae between 09:00 and 13:00 h. As far as we are aware, no time curve of baseline HPI-axis activity has been published in larvae of this age; hence it is difficult to say what the effect of this relatively wide time window is. As sampling was done in the same way for all light regimes and strains, any variation because of this sampling time window is the same across light regimes and strains. Given that we see little variation in the data, we believe that our time window of sampling was adequate.

### Comparison of AB and TL

The present data extend data of earlier studies by showing that light regimes affect gene-expression in larvae, and more importantly so, strengthen the notion of differences between larvae of the AB and TL strain. It has been shown that genetic and behavioural profiles of the same strain may differ between laboratories (Lange et al., 2013; Butler et al., 2015). This could suggest that the present data may reflect local rather than strain differences. However our data collected on AB and TL zebrafish thus far strongly suggest that our AB and TL strains are similar to those of others (van den Bos et al., 2017). Hence, the data presented here likely reflect true strain differences. Whether the differences between AB and TL are the consequence of the mutation in *connexin 41.8*, that in TL leads to spots rather than stripes (Watanabe et al., 2006), or of higher levels of cortisol in AB than TL (larvae: van den Bos et al., 2017; adults: Gorissen et al., 2015) or a combination hereof, needs further study.

The observed differences between AB and TL larvae add to the increasing number of studies on phenotypical differences between these strains in larval (van den Bos et al., 2017; Liu et al., 2015; Gao et al., 2016) and adult stages (Gorissen et al., 2015; Séguret et al., 2016). Such differences affect among others reproducibility of experiments both within and between laboratories.

## MATERIALS AND METHODS

### Breeding, embryos and larvae

In-house bred adult (>6 months) zebrafish of the AB and TL strains from the fish facilities of the Department of Animal Ecology and Physiology (Radboud University, Nijmegen, The Netherlands) were used for egg production. They were kept in recirculation systems (bio-filtered Nijmegen tap water, ∼28°C, pH 7.5-8, conductivity ∼320 microSiemens/cm; Fleuren and Nooijen, Nederweert, The Netherlands) in 2-litre aquaria (approximately 30 fish of mixed sex) under a 14L:10D cycle (lights on from 09:00 h to 23:00 h), and fed twice daily at 09:00 h (*Artemia* sp. and Gemma Micro 300, Skretting, Wincham, Northwich, Cheshire, UK) and 15:00 h (Gemma Micro 300).

Breeding was done as previously described (van den Bos et al., 2017), starting at least one hour after the last feeding of zebrafish (>16:00 h). Eggs, embryos and larvae were kept under different light regimes up to 5 dpf: (i) 14L:10D (lights on: 09:00 h–23:00 h); light phase: 300-350 lux; dark phase; 0 lux; (ii) 24 h light condition (LL); 400-500 lux; (iii) 24 h darkness condition (DD); light impermeable polystyrene box (in a climate-controlled room); 0 lux.

All experiments were carried out in accordance with the Dutch Animals Act (http://wetten.overheid.nl/BWBR0003081/2014-12-18), the European guidelines for animal experiments (Directive 2010/63/EU; http://eur-lex.europa.eu/legal-content/NL/TXT/HTML/?uri=CELEX:32010L0063) and institutional regulations.

### Gene expression analysis

Larvae (5 dpf) were sampled between 09:00 h and 13:00 h (van den Bos et al., 2017). They were deeply anesthetised by placing them in 0.1% (v/v) 2-phenoxyethanol. To obtain sufficient material for analysis, two larvae per sample were transferred to 2-ml Eppendorf tubes containing a plastic grinding ball. Residual medium was removed with a pipette and samples were snap-frozen in liquid nitrogen and stored at −80°C until total RNA extraction.

Total RNA content of each sample was isolated as previously described (van den Bos et al., 2017). The concentration and quality of RNA in each sample were assessed using a nanodrop spectrometer at 260 and 280 nm wavelength (Nanodrop, Wilmington, DE, USA). Isolated RNA was treated with DNase to remove any (genomic) DNA from the sample. 400 ng RNA was transferred into a PCR strip, and DEPC-treated dH_2_O was added to a volume of 8 μl. To this, 2 μl of DNase mix was added, containing 1 μl 10× DNase I reaction buffer and 1 μl (1 U μl^−1^) amplification grade DNase I (both from Invitrogen, Carlsbad, USA). The resulting mix was incubated for 15 min at room temperature. Afterwards, 1 μl 25 mM EDTA was added to stop the DNase reaction and the reaction mix was incubated for 10 min at 65°C and put back on ice.

After the DNase treatment, samples were used to synthesise cDNA by the addition of 1 μl random primers (250 ng μl^−1^), 1 μl 10 mM dNTP mix, 4 μl 5×1st strand buffer, 1 μl 0.1 M DTT, 1 μl RNaseOUT (40 U μl^−1^), 0.5 μl Superscript II (reverse transcriptase) (200 U μl^−1^) (all from Invitrogen, Carlsbad, USA) and 0.5 μl DEPC-treated dH_2_O. The resulting mix was incubated for 10 min at 25°C for annealing of the primers and then 50 min at 42°C for reverse transcription. Hereafter, enzymes were inactivated by incubating samples at 70°C for 15 min. Finally, 80 μl dH_2_O was added to dilute the samples five times for the qPCR reaction.

To analyse relative gene expression in each sample, real-time qPCR was carried out for each gene of interest. For each qPCR reaction, 16 μl PCR mix [containing 10 μl SYBR green mix (2×) (BioRad, Hercules, USA), 0.7 μl forward and reverse gene-specific primer (10 μM)] and 4.6 μl H_2_O were added to 4 μl of cDNA. The qPCR reaction (3 min 95°C, 40 cycles of 15 s 95°C and 1 min 60°C) was carried out using a CFX 96 (BioRad, Hercules, USA) qPCR machine. Analysis of the data was carried out using a normalisation index of two reference genes [*viz. elongation factor 1 alpha* (*elf1a*) and *ribosomal protein L13* (*rpl13*)] according to Vandesompele and colleagues (2002). Primer sequences of genes of interest can be found in van den Bos et al. (2017). Routine quality check procedures were followed with respect to the qPCR; these include, melting curve analyses, no-RT and no-template controls.

### Statistical analysis

Data were analysed using two-way analysis of variance (ANOVA) (factors: strain and light regime) followed by post hoc testing (Tukey HSD for light regimes; Student's *t*-test between strains per light regime) where appropriate.

We assessed interrelationships of transcript abundance levels using PCA with orthogonal rotation (Varimax rotation with Kaiser normalisation) for each strain. In case of missing samples, data were excluded list-wise. The number of retained factors was based on eigenvalues (>1) and visual inspection of the scree plot. The Kaiser-Meyer-Olkin (KMO) measure of sampling adequacy and the Bartlett test of sphericity were done to ensure that data obeyed analysis criteria; both are measures to assess whether the correlation matrix is suited for factor analysis (Budaev, 2010). The Bartlett assesses whether the matrix deviates from an identity matrix (only correlations on the diagonal) by testing whether off-diagonal correlations are not due to sampling error; the KMO compares the observed correlations and partial correlations among the original variables, i.e. it assesses whether variables share a unique variance (Budaev, 2010). Factor scores were saved and used for further analysis. The following factor loading cut-off points were considered: ≤−0.600 or ≥0.600 (Ferguson, 1989; Budaev, 2010). One-way ANOVAs followed by Tukey's HSD were run per strain.

Statistical analyses were done using IBM SPSS version 23 for Windows (IBM, Armonk, NY, USA). Significance was accepted when *P*≤0.05 (two-tailed), unless otherwise stated.

## References

[BIO028969C1] Ahmad F. (2014). Zebrafish Embryos and Larvae as a Complementary Model for Behavioural Research. *PhD Thesis*. University of Leiden, The Netherlands.

[BIO028969C2] Alderman S. L. and Bernier N. J. (2009). Ontogeny of the corticotropin-releasing factor system in zebrafish. *Gen. Comp. Endocrinol.* 164, 61-69. 10.1016/j.ygcen.2009.04.007 19366623

[BIO028969C3] Alsop D. and Vijayan M. M. (2008). Development of the corticosteroid stress axis and receptor expression in zebrafish. *Am. J. Physiol. Regul. Integr. Comp. Physiol.* 294, R711-R719. 10.1152/ajpregu.00671.2007 18077507

[BIO028969C4] Budaev S. V. (2010). Using principal components and factor analysis in animal behaviour research: caveats and guidelines. *Ethology* 116, 472-480. 10.1111/j.1439-0310.2010.01758.x

[BIO028969C5] Butler M. G., Iben J. R., Marsden K. C., Epstein J. A., Granato M. and Weinstein B. M. (2015). SNPfisher: tools for probing genetic variation in laboratory-reared zebrafish. *Development* 142, 1542-1552. 10.1242/dev.118786 25813542 PMC4392598

[BIO028969C6] Chatzopoulou A., Roy U., Meijer A. H., Alia A., Spaink H. P. and Schaaf M. JM. (2015). Transcriptional and metabolic effects of glucocorticoid receptor α and β signaling in zebrafish. *Endocrinology* 156, 1757-1769. 10.1210/en.2014-1941 25756310

[BIO028969C7] Dyer A. H., Vahdatpour C., Sanfeliu A. and Tropea D. (2016). The role of insulin-like growth factor 1 (IGF-1) in brain development, maturation and neuroplasticity. *Neuroscience* 325, 89-99. 10.1016/j.neuroscience.2016.03.056 27038749

[BIO028969C8] Eivers E., McCarthy K., Glynn C., Nolan C. M. and Byrnes L. (2004). Insulin-like growth factor (IGF) signalling is required for early dorso-anterior development of the zebrafish embryo. *Int. J. Dev. Biol.* 48, 1131-1140. 10.1387/ijdb.041913ee 15602699

[BIO028969C9] Eto K., Mazilu-Brown J. K., Henderson-MacLennan N., Dipple K. M. and McCabe E. R. B. (2014). Development of catecholamine and cortisol stress responses in zebrafish. *Mol. Genet. Metab. Rep.* 1, 373-377. 10.1016/j.ymgmr.2014.08.003 27896111 PMC5121345

[BIO028969C10] Ferguson G. A. (1989). *Statistical Analysis in Psychology and Education*. Columbus: McGraw Hill.

[BIO028969C11] Gao Y., Zhang G., Jelfs B., Carmer R., Venkatraman P., Ghadami M., Brown S. A., Pang C. P., Leung Y. F., Chan R. H. M. et al. (2016). Computational classification of different wild-type zebrafish strains based on their variation in light-induced locomotor response. *Comput. Biol. Med.* 69, 1-9. 10.1016/j.compbiomed.2015.11.012 26688204

[BIO028969C12] Gorissen M., Manuel R., Pelgrim T. N. M., Mes W., de Wolf M. J. S., Zethof J., Flik G. and van den Bos R. (2015). Differences in inhibitory avoidance, cortisol and brain gene expression in TL and AB zebrafish. *Genes Brain Behav.* 14, 428-438. 10.1111/gbb.12220 25906812

[BIO028969C13] Guryev V., Koudijs M. J., Berezikov E., Johnson S. L., Plasterk R. H. A., van Eeden F. J. M. and Cuppen E. (2006). Genetic variation in the zebrafish. *Genome Res.* 16, 491-497. 10.1101/gr.4791006 16533913 PMC1457036

[BIO028969C14] Lange M., Neuzeret F., Fabreges B., Froc C., Bedu S., Bally-Cuif L. and Norton W. H. J. (2013). Inter-individual and Inter-strain variations in zebrafish locomotor ontogeny. *PLoS ONE* 8, e70172-e70114 10.1371/journal.pone.0070172 PMC3739779 23950910

[BIO028969C15] Liu Y., Carmer R., Zhang G., Venkatraman P., Brown S. A., Pang C. P., Zhang M., Ma P. and Leung Y. F. (2015). Statistical analysis of zebrafish locomotor response. *PLoS ONE* 10, e0139521 10.1371/journal.pone.0139521 26437184 PMC4593604

[BIO028969C16] McMenamin S. K., Minchin J. E. N., Gordon T. N., Rawls J. F. and Parichy D. M. (2013). Dwarfism and increased adiposity in the gh1 mutant Zebrafish vizzini. *Endocrinology* 154, 1476-1487. 10.1210/en.2012-1734 23456361 PMC3602633

[BIO028969C17] Séguret A., Collignon B. and Halloy J. (2016). Strain differences in the collective behaviour of zebrafish (Danio rerio) in heterogeneous environment. *R. Soc. Open Sci.* 3, 160451 10.1098/rsos.160451 27853558 PMC5098983

[BIO028969C18] Spence R., Gerlach G., Lawrence C. and Smith C. (2008). The behaviour and ecology of the zebrafish, Danio rerio. *Biol. Rev. Camb. Philos. Soc.* 83, 13-34. 10.1111/j.1469-185X.2007.00030.x 18093234

[BIO028969C19] Stewart A. M., Braubach O., Spitsbergen J., Gerlai R. and Kalueff A. V. (2014). Zebrafish models for translational neuroscience research: from tank to bedside. *Trends Neurosci.* 37, 264-278. 10.1016/j.tins.2014.02.011 24726051 PMC4039217

[BIO028969C20] van den Bos R., Mes W., Galligani P., Heil A., Zethof J., Flik G. and Gorissen M. (2017). Further characterisation of differences between TL and AB zebrafish (Danio rerio): Gene expression, physiology and behaviour at day 5 of the larval stage. *PLoS ONE* 12, e0175420 10.1371/journal.pone.0175420 28419104 PMC5395159

[BIO028969C24] Vandesompele J., De Preter K., Pattyn F., Poppe B., Van Roy N., De Paepe A. and Speleman F. (2002). Accurate normalization of real-time quantitative RT-PCR data by geometric averaging of multiple internal control genes. *Genome Biol.* 3, 1-12. 10.1089/zeb.2013.0926 PMC126239 12184808

[BIO028969C21] Villamizar N., Maria Vera L., Foulkes N. S. and Sánchez-Vázquez F. J. (2014). Effect of lighting conditions on zebrafish growth and development. *Zebrafish* 11, 173-181. 10.1089/zeb.2013.0926 24367902 PMC3991973

[BIO028969C22] Watanabe M., Iwashita M., Ishii M., Kurachi Y., Kawakami A., Kondo S. and Okada N. (2006). Spot pattern of leopard Danio is caused by mutation in the zebrafish connexin41.8 gene. *EMBO Rep.* 7, 893-897. 10.1038/sj.embor.7400757 16845369 PMC1559663

[BIO028969C23] Zhu X., Gleiberman A. S. and Rosenfeld M. G. (2007). Molecular physiology of pituitary development: signaling and transcriptional networks. *Physiol. Rev.* 87, 933-963. 10.1152/physrev.00006.2006 17615393

